# A pan-cancer landscape of telomeric content shows that *RAD21* and *HGF* alterations are associated with longer telomeres

**DOI:** 10.1186/s13073-022-01029-7

**Published:** 2022-02-26

**Authors:** Radwa Sharaf, Meagan Montesion, Julia F. Hopkins, Jiarong Song, Garrett M. Frampton, Lee A. Albacker

**Affiliations:** Foundation Medicine Inc, 150 Second Street, Cambridge, MA 02141 USA

**Keywords:** Telomere, Tumor, RAD21, HGF, Breast, TERC, TERT, ATRX, DAXX, ALT

## Abstract

**Background:**

Cancer cells can proliferate indefinitely through telomere maintenance mechanisms. These mechanisms include telomerase-dependent elongation, mediated by *TERT* activation, and alternative lengthening of telomeres (ALT), linked to loss of *ATRX* or *DAXX*.

**Methods:**

We analyzed the telomeric content of 89,959 tumor samples within the Foundation Medicine dataset and investigated the genomic determinants of high telomeric content, linking them to clinical outcomes, when available.

**Results:**

Telomeric content varied widely by disease type with leiomyosarcoma having the highest and Merkel cell carcinoma having the lowest telomeric content. In agreement with previous studies, telomeric content was significantly higher in samples with alterations in *TERC*, *ATRX,* and *DAXX*. We further identified that amplifications in two genes, *RAD21* and *HGF*, were enriched in samples with high telomeric content, which was confirmed using the PCAWG/ICGC dataset. We identified the minimal amplified region associated with high telomeric content for *RAD21* (8q23.1–8q24.12), which excludes *MYC*, and for *HGF* (7q21.11). Our results demonstrated that *RAD21* and *HGF* exerted an additive telomere lengthening effect on samples with existing alterations in canonical genes previously associated with telomere elongation. Furthermore, patients with breast cancer who harbor *RAD21* alterations had poor median overall survival and trended towards higher levels of Ki-67 staining.

**Conclusions:**

This study highlights the importance of the role played by *RAD21* (8q23.1–8q24.12) and *HGF* (7q21.11) in the lengthening of telomeres, supporting unlimited replication in tumors. These findings open avenues for work aimed at targeting this crucial pathway in tumorigenesis.

**Supplementary Information:**

The online version contains supplementary material available at 10.1186/s13073-022-01029-7.

## Background

Infinite proliferative capacity is a hallmark of cancer [1], which requires tumor cells to evolve mechanisms to overcome the problem of telomere shortening. Telomeres are DNA-protein complexes that function to protect the ends of linear chromosomes from damage. They consist of 5–15 kb of repetitive hexamers and shorten in length by an average of 50–150 base pairs with every cell division [2, 3]. If telomeric length falls below a critical threshold, cells go into replicative senescence and can no longer divide [4, 5]. Hence, the length of telomeres ultimately limits the number of times a cell can divide and acts as a powerful tumor suppressor mechanism.

Telomere length may be maintained in cancer cells by different mechanisms, including telomerase-mediated lengthening and alternative lengthening of telomeres (ALT). The former relies on the overexpression of the telomerase enzyme, encoded by *TERT*, and was observed in 85–90% of tumors [6, 7]. By reverse transcribing an RNA template, encoded by *TERC*, telomerase catalyzes the elongation of telomeric DNA using the 3′ end of the chromosome as a primer [8, 9]. ALT, on the other hand, relies on homologous recombination, where portions of the telomeric region are copied over from one chromosome to another [10, 11]. To date, ALT is not fully understood, but it has been associated with loss-of-function mutations in the chromatin remodeling genes *ATRX* and *DAXX* [12]. ALT is prevalent in specific tumor types, such as gliomas and sarcomas [13, 14].

Given the need of cancer cells to extend telomeres, additional undescribed mechanisms likely contribute to telomere lengthening. To interrogate genomic alterations associated with telomeric content, we characterized the telomeric landscape of 89,959 solid tumor samples across the Foundation Medicine dataset, which represents 81 tumor types sequenced on one platform. We then investigated the genomic determinants of high telomeric content across tumor types and focused on genes likely to have a pan-cancer effect by requiring gene-telomeric content associations in multiple tumor types. To assess potential clinical relevance, we compared the survival of patients whose tumors harbored genomic alterations associated with high telomeric content with patients whose tumors lacked these alterations.

## Methods

### Foundation Medicine dataset

The Foundation Medicine dataset comprised 89,959 solid tumor specimens sequenced as a part of routine clinical care. The pathologic diagnosis of each case was first made in the referring center and was then confirmed in our facility (Foundation Medicine, Cambridge MA) on routine hematoxylin and eosin stained slides. All samples contained a minimum of 20% tumor nuclei. Samples represented 81 unique disease groups and the top five disease ontologies present were lung adenocarcinoma, colon adenocarcinoma (crc), breast carcinoma, ovary serous carcinoma, and pancreas ductal adenocarcinoma. This is the main cohort referenced throughout the manuscript, unless otherwise indicated. For 14,074 samples across various tumors in our dataset, additional RNA sequencing data was available and used for the measurement of TERRA levels. Clinical outcomes data was also available for 1164 breast invasive ductal carcinoma samples as detailed below.

### Sample sequencing

Samples were sequenced using a targeted next-generation sequencing assay as previously described (FoundationOne®CDx) [15, 16]. The samples were assayed by adaptor ligation hybrid capture, performed for all coding exons of 309 cancer-related genes plus select introns from 34 genes frequently rearranged in cancer. Sequencing of captured libraries was performed using the Illumina sequencing platform to a mean exon coverage depth for targeted regions of >500X, and sequences were analyzed for genomic alterations, including short variant alterations (base substitutions, insertions, and deletions), copy number alterations (focal amplifications and homozygous deletions), and select gene fusions or rearrangements. For *TERT*, only the mutations in the promoter region were captured.

Germline variants documented in the dbSNP database (dbSNP142) with two or more counts in the ExAC database, or recurrent variants of unknown significance that were predicted by an internally developed algorithm to be germline were removed, with the exception of known driver germline events (e.g., documented hereditary *BRCA1/2* and deleterious *TP53* mutations). Known confirmed somatic alterations deposited in the Catalog of Somatic Mutations in Cancer were highlighted as biologically significant [17]. All inactivating events (i.e., truncations and deletions) in known tumor suppressor genes were also called as significant. To maximize mutation-detection accuracy (sensitivity and specificity) in impure clinical specimens, the test was previously optimized and validated to detect base substitutions at a ≥5% mutant allele frequency (MAF), indels with a ≥10% MAF with ≥99% accuracy, and fusions occurring within baited introns/exons with >99% sensitivity [15]. Throughout the manuscript, “altered” refers to a sample with known or likely pathogenic genetic alterations, whereas “WT” refers to a sample lacking these alterations or containing variants of unknown significance.

### Telomeric content

The telomeric content of samples was determined using TelomereHunter 1.1.0 [18]. This software tool was run using the default parameters and a repeat threshold set to 7 for 49 bp paired-end reads. TelomereHunter extracts telomeric reads that contain seven instances of the four most common telomeric repeat types (TTAGGG, TCAGGG, TGAGGG, and TTGGGG) and determines the telomeric content by normalizing the telomere read count by all reads in the sample with a GC content of 48–52%. For a subset of samples, RNA sequencing was also performed and used for TERRA detection. TelomereHunter was run on RNA bamfiles to count reads containing at least 7 repeats. Similar to telomeric content measurement for DNA, the TERRA content was calculated by normalizing TERRA read counts by the total number of reads with comparable GC content in the sample.

### ICGC/TCGA Pan-Cancer Analysis of Whole Genomes (PCAWG) Consortium dataset

Samples with available telomeric content within the tumor from Supplementary Data 1 of Sieverling et al. were used for this analysis [19]. Classification of samples into *ATRX/DAXX*, *TERTp*, and *TERC* altered groups was given in the TMM_associated_mut column of the same supplementary data file. Through the ICGC data portal (https://dcc.icgc.org/, [20]), CNSM (copy number somatic mutation) data was obtained and used to check for samples with gains in *RAD21* (hg19 - chr8:117858173–117887105) or *HGF* (hg19 - chr7:81331444–81399452) and were grouped accordingly. Disease groups were analyzed to confirm findings from the Foundation Medicine dataset: *RAD21* included Prost-AdenoCA, Breast-AdenoCA, Breast-LobularCA, Breast-DCIS, and Lung-AdenoCA; *ATRX/DAXX* included CNS-GBM, CNS-LGG, and Panc-Endocrine; and *TERTp* included CNS-GBM, CNS-LGG, and Skin-Melanoma samples. Fewer than 10 samples harbored *HGF* alterations and belonged to any of these disease groups: Kidney-RCC, Prost-AdenoCA, or CNS-GBM. Similarly, fewer than 10 samples harbored *TERC* alterations and were classified as either Prost-AdenoCA or Lung-SCC. Thus, *HGF* and *TERC* were not included in this confirmatory analysis.

For expression analysis, we assessed the subset of samples with sequencing-based gene expression results. For *RAD21*, analysis was performed for breast cancer, prostate adenocarcinoma, and lung adenocarcinoma samples (study IDs: BRCA-US, PRAD-US, PRAD-FR, PRAD-CA, LUAD-US, BRCA-KR). For *HGF*, the analysis was done for prostate adenocarcinoma, brain glioblastoma, and kidney clear cell carcinoma (study IDs: PRAD-US, PRAD-FR, PRAD-CA, GBM-US, KIRC-US). Normalized read counts for *RAD21* and *HGF* were pulled from each sample. Using the ICGC_specimen_id as the sample identifier, we then compared between the expression values of *RAD21* of samples previously identified as *RAD21* amplified vs. WT and the expression values of *HGF* amongst previously identified as *HGF* amplified vs. WT. All *RAD21* amplified and *HGF* amplified samples belonged to the prostate adenocarcinoma disease group.

### Clinico-Genomics cohort and survival analysis

The retrospective clinical analysis utilized the nationwide (US-based) Foundation Medicine–Flatiron Health real-world clinico-genomic database (CGDB, data collected through December 31, 2020) which includes electronic health record (EHR)–derived deidentified data for patients in the Flatiron Health database who underwent comprehensive genomic profiling by Foundation Medicine, linked by de-identified deterministic matching [21]. The de-identified patient-level clinical data originated from the electronic health records of approximately 800 sites of care including structured data (e.g., medication orders and administrations, lab tests, diagnostic codes) in addition to unstructured data (e.g., smoking status, histology) collected via technology-enabled chart abstraction from physicians’ notes by trained medical record abstractors who followed prespecified, standardized policies and procedures. Deidentified patient-level genomic data included specimen (e.g., TMB, tumor purity) and genomic (e.g., gene altered, alteration type) data reported by the Foundation Medicine’s comprehensive genomic profiling test. Institutional Review Board approval of the study protocol was obtained prior to study conduct and included a waiver of informed consent based on the observational, non-interventional nature of the study (WCG IRB, Protocol No. 420180044).

The patients included in the clinical analysis were diagnosed with breast invasive ductal carcinoma. Overall survival was calculated from date of metastatic diagnosis based on a composite mortality variable [22]. To account for left truncation, patients were treated as at risk of death only after the later of their first sequencing report date and their second visit in the Flatiron Health network on or after January 1, 2011, as both are requirements for inclusion in the cohort. For the Kaplan–Meier analyses, the log-rank test was used to compare between *RAD21* WT vs. altered. Due to low sample count, survival analysis could not be performed for *HGF* WT vs. altered.

### Statistics

Wilcoxon rank sum was used to test for differences between the two groups. Dunn’s test, a non-parametric pairwise multiple comparisons test based on rank sums, was performed with a Bonferroni correction to assess differences between multiple sample groups. Association with high telomeric content was performed within each disease ontology, with high telomeric content defined as the top quartile and low content defined as the bottom quartile. We then performed a Fisher’s exact test to check for the enrichment of alterations across baited genes in samples with high telomeric content per disease ontology. We plotted the results for the canonical genes *ATRX*, *DAXX*, *TERTp*, and *TERC*, in addition to all gene hits with a corrected *P* value < 0.05 after Bonferroni’s correction, an odds ratio > 1, and enriched in at least 2 unique disease groups. We performed a Fisher’s exact test to check for the enrichment of amplifications across chromosome bands of chr8q in samples with high telomeric content, defined as harboring telomeric content in the top quartile. All statistical analyses were conducted using R (4.0.2) [23]. MEGSA (version beta 2) was used to test for mutual exclusivity [24]. To denote significance, * represents *p*<0.05, ** *p*<0.01, *** *p*<0.001, and **** *p*<0.0001.

## Results

### Telomeric content across FMI’s cohort

**Fig. 1:**
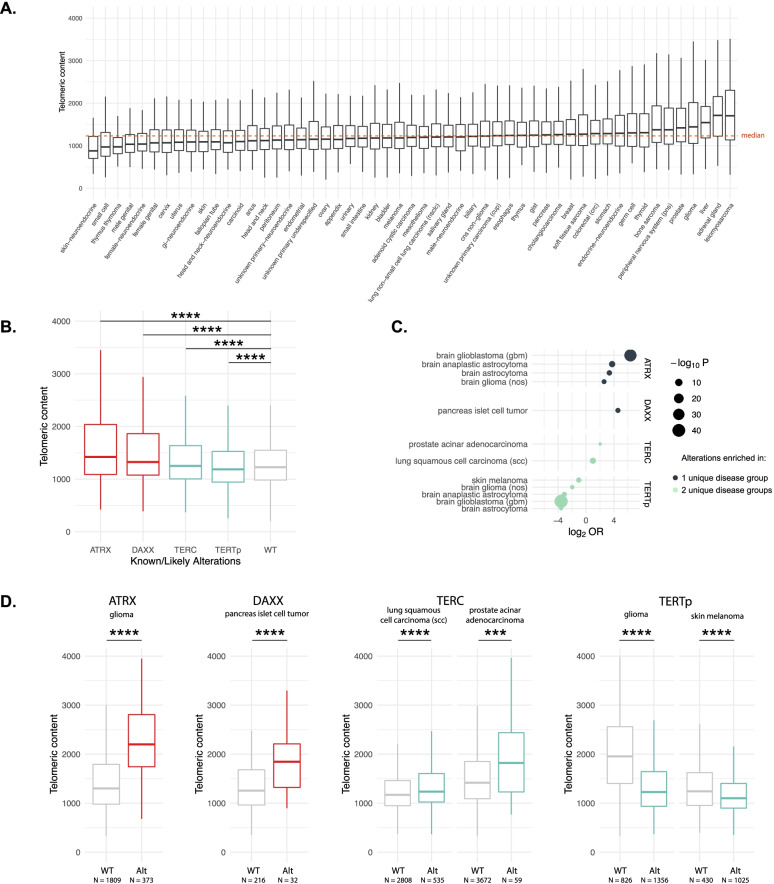
Landscape of telomeric content across diseases and impact of alterations in canonical telomere elongation genes. **A** Boxplot depicting the telomeric content of samples within each disease group. Analysis was restricted to disease groups with more than 40 samples. The median telomeric content of all samples is shown as an orange dotted line. **B** Boxplot showing the telomeric content of samples with pathogenic alterations in *ATRX*, *DAXX*, *TERC*, and *TERTp* or are non-altered (WT). Only comparisons against WT are shown. **** signifies *p*<0.0001. **C** A plot showing disease groups where *ATRX*, *DAXX*, *TERC*, and *TERTp* alterations were associated with significant differences in telomeric content. Per gene and tumor type, we compared the frequency of alterations within samples in the top quartile of telomeric content to the frequency of alterations in the bottom quartile. The size of each circle represents the -log_10_ *P* value and its position along the *x*-axis represents the log_2_ odds ratio. Genes with a signal in one unique disease group are shown in navy blue, while those with signals in two unique disease groups are colored in mint green. *P* values were adjusted for multiple hypothesis testing using Bonferroni’s correction. OR, odds ratio. **D** Boxplots showing the impact of alterations within *ATRX*, *DAXX*, *TERC*, and *TERTp* on the telomeric content within select diseases are shown. Diseases were selected based on the results shown in **C**. WT, wild-type; Alt, altered. *** signifies *p*<0.001 and **** *p*<0.0001

We assessed the telomeric content of 89,959 unique tumor samples from the Foundation Medicine dataset across 81 unique disease groups using TelomereHunter [18] (Additional file 1: Table S1). The median telomeric content of samples varied across disease groups, ranging from 1766.0 TRPM (telomeric reads per GC content-matched million reads) in leiomyosarcoma to 880.1 TRPM in neuroendocrine tumors of the skin (Merkel cell carcinoma) (Fig. 1A and Additional file 1: Table S1). When we excluded samples altered in *ATRX* or *DAXX* to remove ALT, the range dropped from 885.9 to 835.5. In agreement with previous studies [19, 25], median telomeric content in pediatric tumor samples (<18 years old) was significantly higher than samples from older age groups (1337.8 vs. 1229.4 TPRM, Additional file 2: Fig. S1). Diseases with a high proliferative index [26], such as cervical and lung squamous cell carcinoma, trended towards low telomeric content, while slowly proliferating diseases, such as gliomas, generally had high median telomeric content. Moreover, we observed that sarcomas, such as gastrointestinal stromal tumors (GIST), soft tissue sarcoma, leiomyosarcoma, and bone sarcoma, tend to have high telomeric content, while neuroendocrine tumors from many anatomic sites including the skin, female reproductive tract, and gastrointestinal tract harbored low telomeric content, with a notable exception of pancreatic islet cell tumors (endocrine-neuro group), which harbor a high level of ALT-related genetic alterations and display high telomeric content (Additional file 2: Fig. S2).

### Impact of alterations in ATRX/DAXX/TERTp/TERC on telomeric content

Next, we assessed genes known to modulate telomeric content and confirmed that samples with alterations in *ATRX*, *DAXX*, or *TERC* were associated with significantly greater telomeric content (1470, 1350, 1257 median TRPM) compared to wild-type (WT) samples (1230 median TRPM), while those with *TERT* promoter (*TERTp*) mutations were associated with lower telomeric content (1188 median TRPM, *p*<0.0001 for all, Fig. 1B, Additional file 1: Table S2) [19, 27]. The impact of *ATRX* and *DAXX* alterations on telomeric content was proportional to tumor purity (Additional file 2: Fig. S3). We also investigated tumor-specific trends in these genes by comparing the frequency of alterations within samples in the top quartile of telomeric content compared to the bottom quartile for each tumor type (Additional file 1: Table S3). In gliomas, alterations in *ATRX* were significantly enriched in the top quartile (Bonferroni-corrected *p*<0.0001, odds ratio (OR) = 64, Fig. 1C, D, Additional file 1: Table S3 and S4). Alterations in *DAXX* were significantly enriched in the top quartile of pancreatic islet cell tumors (Bonferroni-corrected *p*<0.01, OR = 23.9), and *TERC* alterations were significantly enriched in the top quartile of prostate acinar carcinoma (Bonferroni-corrected *p*<0.05, OR = 4.1) and lung squamous cell carcinoma (Bonferroni-corrected *p*<0.0001, OR = 2.0, Fig. 1C, D, Additional file 1: Table S3 and S4). A summary of all alterations seen in these four genes is provided in Additional file 1: Table S5. Thus, our assessment of telomeric content is consistent with previously published studies on *ATRX*, *DAXX*, *TERC*, and *TERTp* [19, 27].

### Impact of RAD21 and HGF on telomeric content

**Fig. 2:**
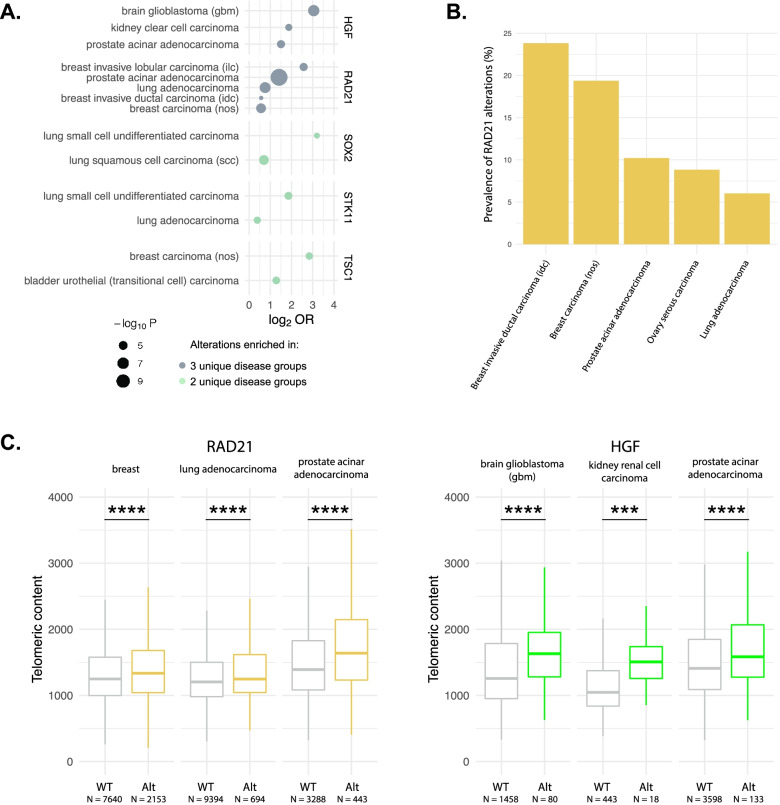
Impact of alterations in *RAD21* and *HGF* on telomeric content in FMI dataset. **A** A plot showing genes where alterations were associated with significant differences in telomeric content within a disease group. Per gene and tumor type, we compared the frequency of alterations within samples in the top quartile of telomeric content to the frequency of alterations in the bottom quartile. The size of each circle represents the -log_10_ *P* value and its position along the *x*-axis represents the log_2_ odds ratio. Genes with a signal in 3 unique disease groups are shown in slate blue, while those with signals in two unique disease groups are colored in mint green. *P* values were adjusted for multiple hypothesis testing using Bonferroni’s correction. OR, odds ratio. **B** Bar plot depicting the prevalence of *RAD21* alterations within the top 5 disease ontologies. **C** Boxplots showing the impact of alterations within *RAD21* and *HGF* on the telomeric content of samples in the FMI dataset. Diseases were selected based on the results shown in **A**. WT, wild-type; Alt, altered. *** signifies *p*<0.001 and **** *p*<0.0001

Having confirmed known genomic associations with telomeric content, we assessed 324 genes in the FMI gene panel for an association with high telomeric content within each disease. Alterations in *RAD21* and *HGF* were each enriched in high telomeric content samples derived from 3 unique disease groups (Fig. 2A, Bonferroni-corrected *p*<0.01 for all and Additional file 1: Table S3). Alterations in *RAD21* were enriched in high telomeric content samples from three breast cancer histologies, breast invasive lobular carcinoma (OR = 5.9), breast invasive ductal carcinoma (OR = 1.5), and breast carcinoma (nos) (OR = 1.5, Fig. 2A), as well as prostate acinar adenocarcinoma (OR = 2.7), and lung adenocarcinoma (OR = 1.7). These diseases represent four of the top five in terms of prevalence of *RAD21* alterations (Fig. 2B, Additional file 1: Table S6). Alterations in *HGF* were significantly enriched in high telomeric content samples from kidney clear cell carcinoma (OR = 3.7), brain glioblastoma (OR = 8.3), and prostate acinar adenocarcinoma (OR = 2.8, Fig. 2A). Median telomeric content in samples with alterations in *RAD21* was significantly higher than in WT for breast tumors, lung adenocarcinoma, and prostate acinar adenocarcinoma (Fig. 2C, Additional file 1: Table S4, *p*<0.0001 for all). In addition, median telomeric content of *HGF* altered samples was significantly higher in brain glioblastoma, kidney clear cell carcinoma, and prostate acinar adenocarcinoma (Fig. 2C, Additional file 1: Table S4, *p*<0.001 for all). A summary of all alterations seen in *RAD21* and *HGF* is provided in Additional file 1: Table S5.

**Fig. 3:**
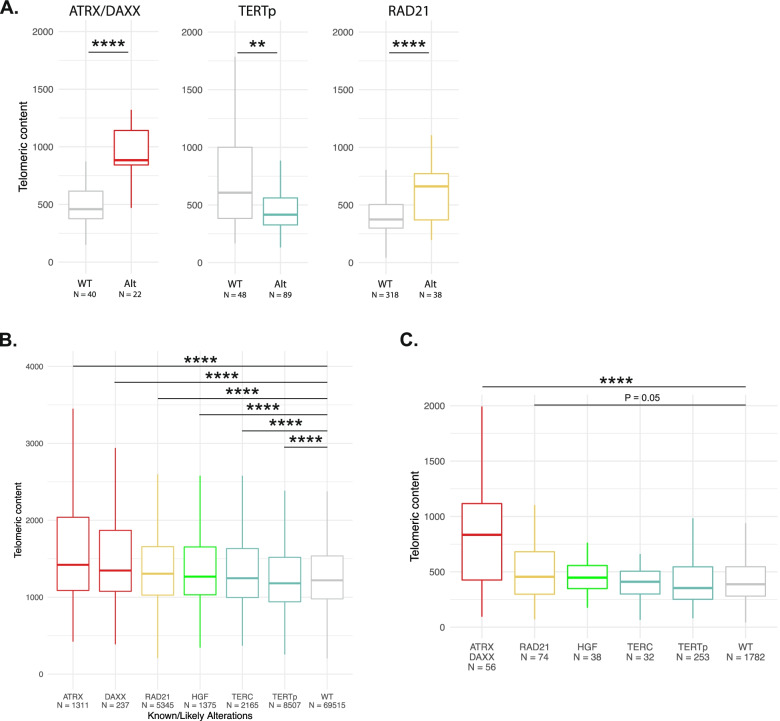
Impact of alterations on telomeric content in PCAWG dataset and pan-cancer. **A.** Boxplots showing the impact of alterations within *ATRX*/*DAXX*, *TERTp*, and *RAD21* on the telomeric content of samples in the PCAWG dataset. Diseases were selected based on the results shown in Figs. 1C and 2A. For *RAD21*, Prost-AdenoCA, Breast-AdenoCA, Breast-LobularCA, Breast-DCIS, and Lung-AdenoCA were included. For *ATRX/DAXX*, CNS-GBM, CNS-LGG, and Panc-Endocrine were included and for *TERTp*, the analysis included CNS-GBM, CNS-LGG, and Skin-Melanoma samples. WT, wild-type; Alt, altered. ** signifies *p*<0.01 and **** *p*<0.0001. Boxplots showing the impact of alterations in *ATRX*, *DAXX*, *RAD21*, *HGF*, *TERC*, and *TERTp* on telomeric content of all samples within the FMI dataset (**B**) and the PCAWG dataset (**C**). For **B** and **C**, all samples with available telomeric content measurements were included regardless of their disease ontology. Dunn’s test, a non-parametric pairwise multiple comparisons test based on rank sums, was performed with a Bonferroni correction to assess differences between multiple sample groups. Only comparisons against WT are shown. **** signifies *p*<0.0001

To validate our findings, we analyzed the telomeric content of 2519 samples from the ICGC/TCGA Pan-Cancer Analysis of Whole Genomes (PCAWG) Consortium [19]. We observed that samples within disease groups identified in our previous analysis (Prost-AdenoCA, Breast-AdenoCA, Breast-LobularCA, Breast-DCIS, and Lung-AdenoCA) and harboring amplifications in *RAD21* had significantly higher telomeric content compared to WT samples (Fig. 3A). *HGF* was not evaluable due to low sample numbers harboring an *HGF* amplification and belonging to one of the following disease groups, Kidney-RCC, Prost-AdenoCA, or CNS-GBM. We also assessed the relationship between amplification and expression of *RAD21* and *HGF*. Median expression of *RAD21* in *RAD21* amplified samples was higher than in WT samples (Additional file 2: Fig. S4; *RAD21*: 1748.7, WT: 1233.8; *p*<0.05), although the sample size was small with only four *RAD21* amplified samples. For *HGF*, there were 5 *HGF* amplified samples with available expression values for *HGF* (median: 32.3) vs. 66 WT samples (Additional file 2: Fig. S4, median: 29.2, *p*>0.05). We also performed a pan-cancer analysis of telomeric content. Samples from the FMI dataset with alterations in *ATRX*, *DAXX*, *RAD21*, *HGF*, or *TERC* had higher telomeric content than WT (Fig. 3B, Additional file 1: Table S7). The pan-cancer PCAWG dataset had a similar pattern as the FMI dataset, where both the *ATRX*/*DAXX* and *RAD21* altered groups had higher telomeric content compared to WT samples (Fig. 3C). Prevalence of *RAD21* alterations varied widely across disease ontologies and tended to mostly occur in diseases with low rates of alterations in the other telomere maintenance genes (Additional file 2: Fig. S5). Thus, our results identified an association between alterations in *RAD21* and high telomeric content pan-cancer, as well as in specific disease groups, in both the FMI and PCAWG datasets.

### Genetic alterations co-occurring with RAD21 and HGF alterations

**Fig. 4:**
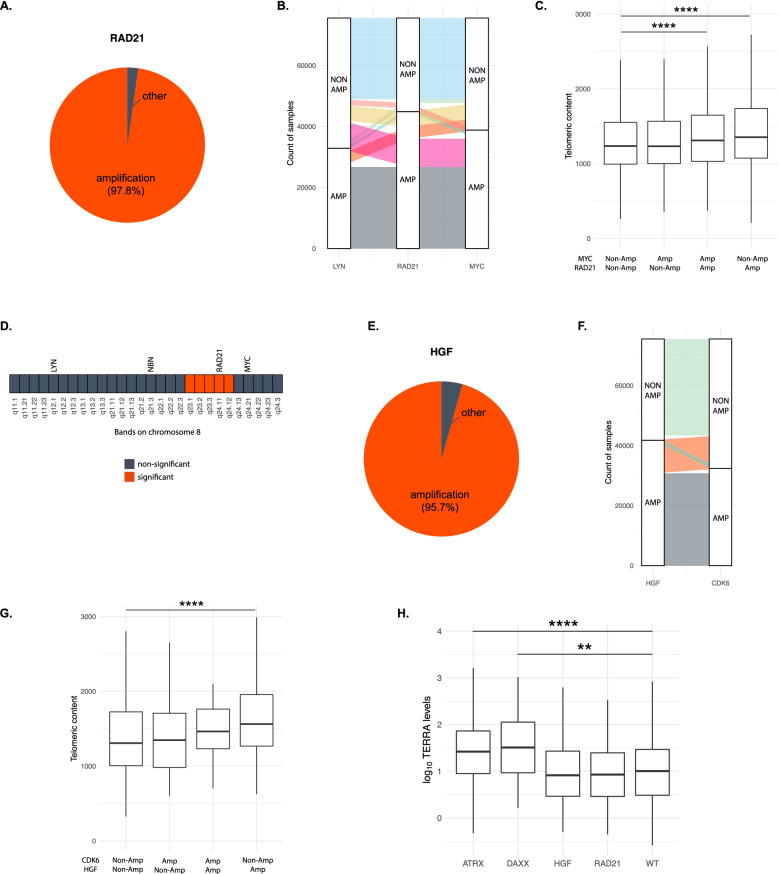
Impact of amplifications in *RAD21* and *HGF* on telomeric content. **A** Pie chart showing the prevalence of amplifications across all *RAD21* alterations within prostate acinar adenocarcinoma, lung adenocarcinoma, breast carcinoma (nos), breast invasive lobular carcinoma, and breast invasive ductal carcinoma. **B** Alluvial plot depicting count of samples with co-amplifications in *LYN*, *RAD21*, and *MYC* within prostate acinar adenocarcinoma, lung adenocarcinoma, breast carcinoma (nos), breast invasive lobular carcinoma, and breast invasive ductal carcinoma samples. Colors of the streams were used to distinguish between the groups. AMP, amplified, NON AMP, non-amplified. **C.** Boxplot showing the impact of *RAD21* and/or *MYC* amplifications on telomeric content of samples pan-cancer. **** denotes p<0.0001. **D** Analysis of enrichment for high telomeric content in samples with amplifications across chromosome bands in chr8q. **E** Pie chart showing the prevalence of amplifications within all *HGF* alterations within brain glioblastoma, prostate acinar adenocarcinoma, and kidney clear cell carcinoma. **F** Alluvial plot depicting count of samples with co-amplifications in *HGF* and *CDK6* within brain glioblastoma, prostate acinar adenocarcinoma, and kidney clear cell carcinoma. AMP, amplified; NON-AMP, non-amplified. **G** Boxplot showing the impact of amplifications of *HGF* and/or *CDK6* on telomeric content of samples pan-cancer. **** denotes p<0.0001. **H** Boxplot showing log_10_ TERRA levels in samples with alterations in *ATRX*, *DAXX*, *HGF*, *RAD21*, and those lacking alterations in any one of these genes (WT). Only comparisons against WT are shown. ** denotes *p*<0.01 and **** denotes *p*<0.0001. TERRA, Telomeric Repeat-containing RNA

Within breast, prostate, and lung tumors, 97.8% of the identified *RAD21* alterations were copy number amplifications (Fig. 4A, Additional file 1: Table S8). *RAD21* lies on chr8q, 10 Mb away from *MYC* and 60 Mb away from *LYN*. *MYC* is co-amplified in 80.2% of samples with *RAD21* amplifications and *LYN* is co-amplified in 68.3% of them (Fig. 4B). To discern whether *MYC* amplifications were a confounding factor in our analysis, we assessed telomeric content based on *RAD21* and *MYC* status. Elevated telomeric content was only associated with *RAD21* amplification and not *MYC* amplification (Fig. 4C). We also assessed the minimal amplified region on chr8q which had an association with high telomeric content. Our analysis showed that samples with amplifications in the region encompassing q23.1–q24.12 on chr8q have significantly higher telomeric content (Fig. 4D, Additional file 1: Table S9). *HGF* alterations were classified as amplifications in 95.7% of kidney, brain, and prostate tumors (Fig. 4E, Additional file 1: Table S8). In samples with an *HGF* amplification, *CDK6* was amplified in 73.6% of samples (Fig. 4F). Samples with only an *HGF* amplification had significantly higher telomeric content compared to WT samples (Fig. 4G). Analysis of the minimally amplified region of *HGF* showed only the amplification of the *HGF* gene itself was significantly associated with higher telomeric content (data not shown). While it can be difficult to identify the causative gene in a copy number amplification in cancer, we show that high telomeric content is associated with *RAD21* and *HGF* but not nearby oncogenes, and furthermore, we identify a minimal amplified interval around *RAD21* consisting of 8q23.1–q24.12.

### Impact of RAD21 and HGF alterations on TERRA levels

Mammalian telomeres are transcribed into long noncoding telomeric repeat-containing RNA (TERRA), which localize to telomeres and regulate telomerase activity. Telomere elongation via the alternative lengthening of telomeres (ALT) mechanism has been linked to elevated levels of TERRA [19, 27, 28]. Thus, to check if *RAD21* or *HGF* alterations are associated with ALT, we measured TERRA levels from RNA sequencing data, available for 14,074 samples originating from various disease ontologies using TelomereHunter [18]. As expected, samples with *ATRX* or *DAXX* alterations expressed significantly higher levels of TERRA compared to WT (*p*<0.0001 and *p*<0.01, respectively, Fig. 4H, Additional file 1: Table S10). TERRA levels in *RAD21* and *HGF* altered samples did not significantly differ from WT samples (Fig. 4H). Thus, we found no evidence of an ALT-based mechanism in samples with *RAD21* or *HGF* alterations, which suggests that a conventional mechanism utilizing *TERT* may be responsible for increased telomeric content in these samples.

### Additive effect of RAD21 and HGF

**Fig. 5:**
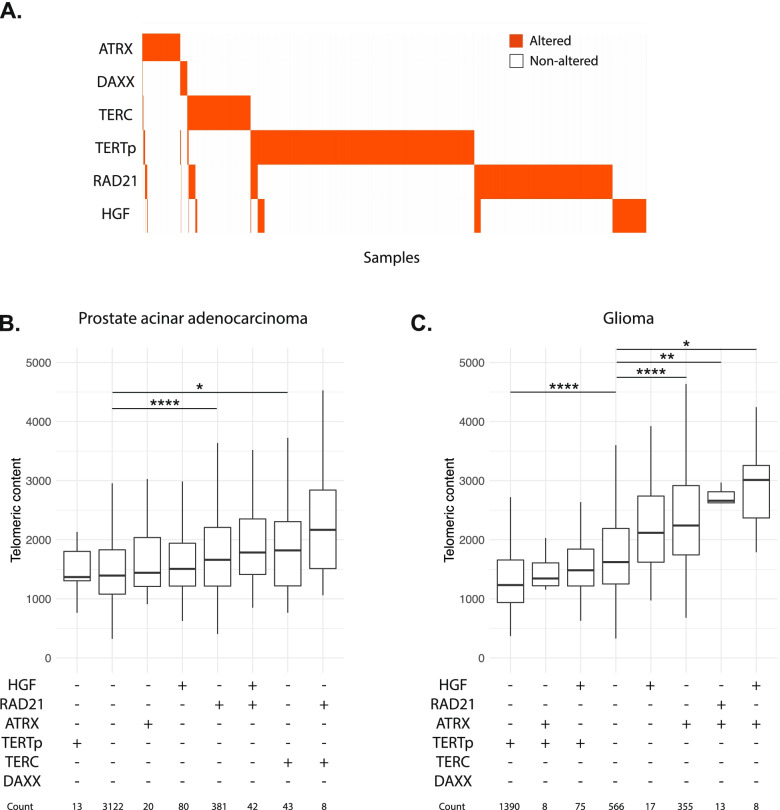
Mutual exclusivity of telomere-maintenance genes and impact of co-occurrence when it rarely occurs. **A** Tile plot showing the distribution of alterations in *ATRX*, *DAXX*, *TERC*, *TERTp*, *RAD21*, and *HGF* within 20,444 samples in the FMI dataset. Plot depicts samples with at least one alteration in these genes. Altered samples are shown in orange and non-altered samples are shown as white. The impact of multiple alterations within these genes on telomeric content is shown for prostate acinar adenocarcinoma in **B** and gliomas in **C**. Within each group, the symbol (+) means altered and the symbol (−) means non-altered. * denotes *p*<0.05, ** *p*<0.01, *** *p*<0.001, and **** *p*<0.0001

Alterations within *RAD21* were predominantly mutually exclusive with alterations in the four canonical genes known to impact telomeric content, *ATRX*/*DAXX*/*TERTp*/*TERC* (*p*=7E-73), suggestive of an independent mechanism of telomere maintenance exerted by RAD21 in these single altered samples (Fig. 5A). In rare samples with multiple alterations, telomeric content of the double altered group was higher than that of the single altered groups in both prostate acinar adenocarcinoma (Fig. 5B, Additional file 1: Table S11) and in glioma (Fig. 5C, Additional file 1: Table S11). Three out of the top four groups ordered by telomeric content had at least one alteration in *RAD21* or *HGF* or both, suggesting an additive independent effect of these alterations on telomeric content and lack of redundancy with alterations in the canonical genes *ATRX*, *DAXX*, *TERTp*, and *TERC*.

### Impact of RAD21 alterations on overall survival in breast

**Fig. 6:**
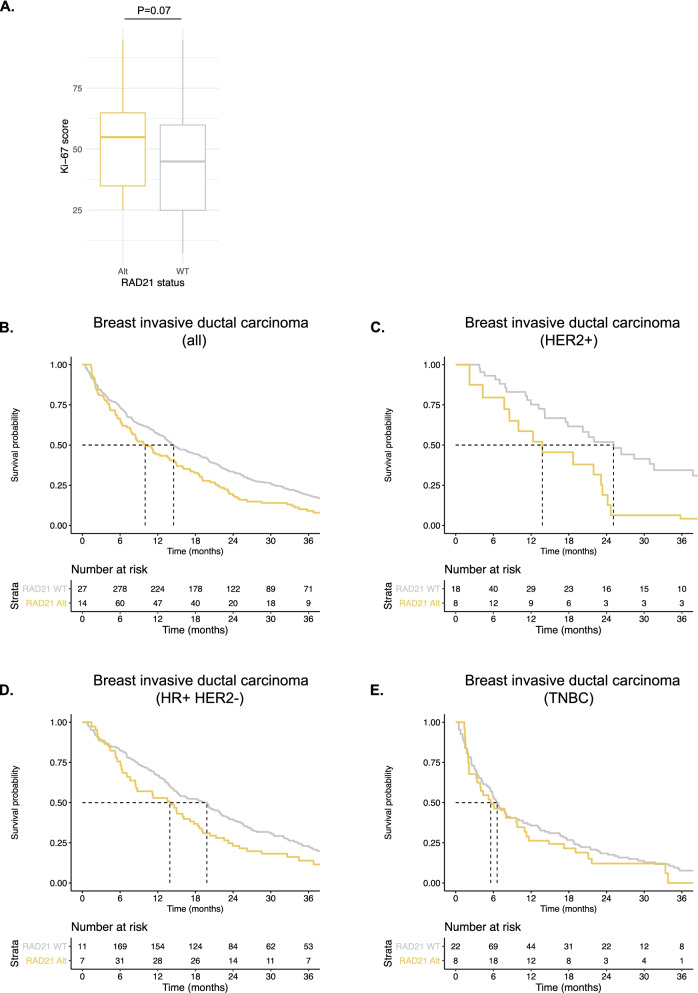
Survival analysis of *RAD21* altered vs. non-altered breast tumors. **A.** Boxplot showing the Ki-67 score of *RAD21* altered vs. non-altered breast samples. Kaplan-Meier curves showing the effect of *RAD21* alterations on the survival probability of all breast invasive ductal carcinoma samples (**B**), HER2+ samples (**C**), HR+ HER2- samples (**D**), and TNBC samples (**E**). Significance was determined by log-rank test. The table underneath each plot shows the number of subjects at risk. HR, hormone receptor; WT, wild-type; Alt, altered

Given the positive association between *RAD21* alterations and telomeric content, we hypothesized that *RAD21* alterations would negatively impact the median overall survival (mOS) of patients with cancer. We assessed a real-world clinico-genomics cohort of 1164 breast invasive ductal carcinomas in which 20.6% (240) of patient tumors harbored a *RAD21* alteration and 79.4% (924) were *RAD21* WT. In a subset of 74 samples with Ki-67 staining, the median Ki-67 score of *RAD21* alt samples was 65% compared to 35% in the *RAD21* WT group, which trended towards statistical significance (Fig. 6A, *p*=0.07). Patients with *RAD21* alt tumors had a significantly decreased mOS compared to the *RAD21* WT group (Fig. 6B; mOS (months) *RAD21* alt: 10.0 [7.7–14.9]; *RAD21* WT: 14.5 [13.0–18.3]; HR *RAD21* alt = 1.3 [1.1–1.7]; *p* = 0.01). Furthermore, we performed a subgroup analysis for HER2+ tumors, HR+ (hormone receptor positive) HER2− tumors, and triple-negative breast cancer (TNBC). Patients with *RAD21* alt HER2+ tumors had a significantly decreased mOS compared to the WT group (Fig. 6C; mOS (months) *RAD21* alt: 13.8 [7.7–24.2]; *RAD21* WT: 25.1 [17.8–38.8]; HR *RAD21* alt = 2.1 [1.1–3.8]; *p* = 0.03). For HR+ HER2− tumors, there was a trend towards lower mOS for *RAD21* alt compared to the WT group (Fig. 6D; mOS (months) *RAD21* alt: 13.9 [8.4–18.6]; *RAD21* WT: 19.8 [15.0–22.1]; HR *RAD21* alt = 1.2 [0.9–1.6], *p* = 0.22). No differences were observed between the two groups within TNBC (Fig. 6E; mOS *RAD21* alt: 5.6 [2.2–11.3]; *RAD21* WT: 6.6 [5.1–10.1]). In total, *RAD21* alterations were associated with a worse mOS in the total breast invasive ductal carcinoma cohort, with the strongest effect observed in the HER2 positive subset.

## Discussion

In this study, we investigated the genomic determinants of high telomeric content across 89,959 tumor samples in the Foundation Medicine dataset and found that samples with high telomeric content were enriched in amplifications in *RAD21* (8q23.1–8q24.12) or *HGF* (7q21.11) pan-cancer and within select disease ontologies. Furthermore, *RAD21* alterations were negatively associated with median overall survival in breast cancer.

Our results for *ATRX*, *DAXX*, *TERTp*, and *TERC* are in agreement with previously published results in the PCAWG [19] and TCGA datasets [27]. Alterations in *ATRX* and *DAXX* were observed in 1.7% of our cohort, while alterations in the *TERT* promoter (*TERTp*) and *TERC* were observed in 11.9% of our cohort. Additionally, we found that alterations in *RAD21*, observed in 5.9% of our cohort, were significantly associated with greater telomeric content in prostate, breast, and lung tumors. We confirmed this finding using the publicly-available PCAWG/ICGC dataset and published telomeric content values [19, 20]. *RAD21* has been shown to be important for telomere end protection [29, 30] and its depletion prevented alternative lengthening of telomeres (ALT) in zebrafish brain tumor cells in vivo [31]. In our dataset, we found that among the top five disease ontologies in terms of *RAD21* alteration prevalence, three were breast cancer histologies and one was prostate. It was demonstrated that *RAD21* is overexpressed in 80% of breast cancer cell lines [32], in 30–40% of hormone-refractory prostate cancers and xenografts [33]. Furthermore, alterations in *HGF*, observed in 1.5% of our cohort, were significantly associated with greater telomeric content in kidney, brain, and prostate tumors. It was previously reported that *HGF* increases telomerase activity in vitro [34, 35] and treatment with HGF was shown to increase telomere length [36].

Mechanistically, amplifications may increase gene expression to mediate oncogenesis. We showed anecdotal evidence from the PCAWG dataset that *RAD21* amplified samples had increased expression. A review of TCGA data presented in cBioportal for breast, lung, and prostate cancers showed that *RAD21* expression correlated with GISTIC copy number assessments. These data are suggestive that *RAD21* amplification is related to increased expression. However, *HGF* amplifications were not associated with increased expression in the PCAWG dataset or TCGA data. Multiple regulatory mechanisms may act on growth factors; thus, the connection between *HGF* amplification and increased telomere content may be indirect.

Overall, we found that alterations within *RAD21*, *ATRX*, *DAXX*, *TERTp*, and *TERC* were predominantly mutually exclusive, which is a frequent feature of genes that affect the same process [37–39]. Of note, 1.7% of samples harbored alterations in multiple genes. In vitro experiments have shown that telomere maintenance mechanisms could in principle coexist within the same tumor cells [40–42] and this was observed in some clinical specimens [43, 44]. In our dataset, samples with an additional alteration in *RAD21* or *HGF* on top of one of the canonical genes had higher telomeric content, displaying their non-redundant impact on telomere maintenance.

Since our assay only captures certain regions of the genome, our analysis was limited to genes baited in Foundation Medicine’s testing. Amplifications present an additional challenge in assigning causality to a specific gene since many genes can be contained within an amplified region. We assessed amplified segments for association with elevated telomeric content and found a significant association with the region around *RAD21* from 8q23.1 to 8q24.12. While further studies will be required to mechanistically link amplification of *RAD21* to longer telomeres, the region identified does exclude *MYC* and *LYN,* oncogenes that are also on chr8q. Furthermore, our testing detects mutations only in the *TERT* promoter and doesn’t capture other mechanisms that could lead to telomerase overexpression, including gene amplification of *TERT*, enhancer hijacking, epigenetic alteration of repressor elements, activation of transcription factors, among others [45–47]. Overall, 77.3% of samples in our cohort were WT for *ATRX*, *DAXX*, *TERTp*, *TERC*, *RAD21*, and *HGF*. Future studies are needed to elucidate the telomere maintenance mechanisms in these samples.

We also investigated the prognostic impact of *RAD21* alterations. Previously, it was reported that *RAD21* overexpression is a marker for poor prognosis in breast [48–50], bladder [51], *KRAS* mutant colorectal carcinomas [52], and NSCLC [53]. Our results are in line with this finding, where patients with *RAD21* altered breast invasive ductal carcinoma had significantly worse median overall survival compared to their WT counterparts. These results suggest that *RAD21* alterations, via promoting telomere elongation, enable tumor cells to continue replicating, thus leading to poor overall survival. Our conclusion is further supported by the observation that Ki-67 staining, a marker of cell proliferation, trended higher in the *RAD21* altered group compared to the WT group. Multiple groups have shown that patients with high Ki-67 staining displayed worse overall survival than those with low Ki-67 staining levels [54–57], likely due to the higher rate of tumor cell replication.

## Conclusions

In this report, we analyzed the telomeric content of samples across the Foundation Medicine dataset. In addition to the well-established role of *ATRX*, *DAXX*, and *TERC* in telomere elongation, we also found an enrichment of *RAD21* and *HGF* amplifications in samples with high telomeric content. In addition, *RAD21* altered breast patients have a worse median overall survival compared to *RAD21* WT. Our findings extend our current understanding of the biology of telomere maintenance mechanisms within cancer.

## Supplementary Information

**Additional file 1: Table S1.** Sample counts and median telomeric content per disease group. **Table S2.** Telomeric content of samples (pan-cancer) with alterations in *ATRX*, *DAXX*, *TERC*, *TERTp,* or WT. **Table S3.** Hits from enrichment analysis of high telomeric content samples with genetic alterations per disease ontology. **Table S4.** Telomeric content of samples (within specific disease ontologies) with alterations in *ATRX*, *DAXX*, *TERC*, *TERTp, RAD21,* or *HGF* compared to WT. **Table S5.** In each tab, a summary of alterations observed in *ATRX*, *DAXX*, *TERC*, *TERTp*, *RAD21*, and *HGF* is provided. SV, short variant alteration; CN, copy number alteration; RE, rearrangement. For SVs, the protein effect is listed and for REs, the partner gene is noted. **Table S6.** Top five disease ontologies in terms of *RAD21* alteration prevalence. **Table S7.** Telomeric content of samples (pan-cancer) with alterations in *ATRX*, *DAXX*, *TERC*, *TERTp, RAD21, HGF,* or WT. **Table S8.** Count of samples with alterations vs. amplifications in *RAD21* and *HGF*. **Table S9.** Hits from enrichment analysis of high telomeric content samples in amplifications across chr8q chromosome bands. **Table S10.** Levels of TERRA in samples (pan-cancer) with alterations in *ATRX*, *DAXX*, *RAD21*, *HGF,* or WT. **Table S11.** Telomeric content of samples with multiple alterations in *ATRX*, *DAXX*, *TERC*, *TERTp, RAD21,* or *HGF* (within specific disease ontologies).

**Additional file 2: Fig. S1.** Telomeric content within age groups. Boxplot showing the telomeric content of samples by age group. Analysis was restricted to samples that are non-altered in *ATRX*, *DAXX*, *TERTp*, *TERC*, *RAD21*, and *HGF*. **** denotes *p*<0.0001. **Fig. S2.** Frequency of genetic alterations across disease groups of neuroendocrine tumors. gi, gastrointestinal. **Fig. S3.** Impact of tumor purity on telomeric content. Boxplot showing the telomeric content of samples with alterations in *ATRX*, *DAXX* and *TERTp* across different tumor purities. *** denotes *p*<0.001 and **** denotes *p*<0.0001. **Fig. S4.** Expression levels of RAD21 (A) and HGF (B) in PCAWG dataset. Plot showing expression levels of the indicated genes. Each dot is representative of one sample. * denotes *p*<0.05. **Fig. S5.** Frequency of alterations across disease ontologies. Barplot showing the percentage of samples with alterations in *ATRX*, *DAXX*, *TERC*, *TERTp*, *RAD21*, and *HGF* across disease ontologies. Analysis was restricted to disease ontologies with more than 40 samples.

## Data Availability

Academic researchers can gain access to Foundation Medicine data in this study by contacting the corresponding author and filling out a study review committee form. You and your institution will be required to sign a data transfer agreement. Survival data that support the findings of this study have been originated by Flatiron Health, Inc. and Foundation Medicine, Inc. These de-identified data may be made available upon request and are subject to a license agreement with Flatiron Health and Foundation Medicine; interested researchers should contact cgdb-fmi@flatiron.com to determine licensing terms.

## Abbreviations

ALT: Alternative lengthening of telomeres
TMM: Telomere maintenance mechanisms
TCGA: The Cancer Genome Atlas
PCAWG: Pan-Cancer Analysis of Whole Genomes
ICGC: International Cancer Genome Consortium
mOS: Median overall survival
OR: Odds ratio
