# Supplementary material for: A pan-cancer landscape of telomeric content shows that RAD21 and HGF alterations are associated with longer telomeres
Source: Genome Med. 2022 Feb 26;14:25. doi: 10.1186/s13073-022-01029-7 (PMC8883689; doi:10.1186/s13073-022-01029-7)
Supplement: Supplementary file 2 — Additional file 2: Fig. S1. Telomeric content within age groups. Boxplot showing the telomeric content of samples by age group. Analysis was restricted to samples that are non-altered in ATRX, DAXX, TERTp, TERC, RAD21, and HGF. **** denotes p<0.0001. Fig. S2. Frequency of genetic alterations across disease groups of neuroendocrine tumors. gi, gastrointestinal. Fig. S3. Impact of tumor purity on telomeric content. Boxplot showing the telomeric content of samples with alterations in ATRX, DAXX and TERTp across different tumor purities. *** denotes p<0.001 and **** denotes p<0.0001. Fig. S4. Expression levels of RAD21 (A) and HGF (B) in PCAWG dataset. Plot showing expression levels of the indicated genes. Each dot is representative of one sample. * denotes p<0.05. Fig. S5. Frequency of alterations across disease ontologies. Barplot showing the percentage of samples with alterations in ATRX, DAXX, TERC, TERTp, RAD21, and HGF across disease ontologies. Analysis was restricted to disease ontologies with more than 40 samples. [file 13073_2022_1029_MOESM2_ESM.pptx]

## Slide 1
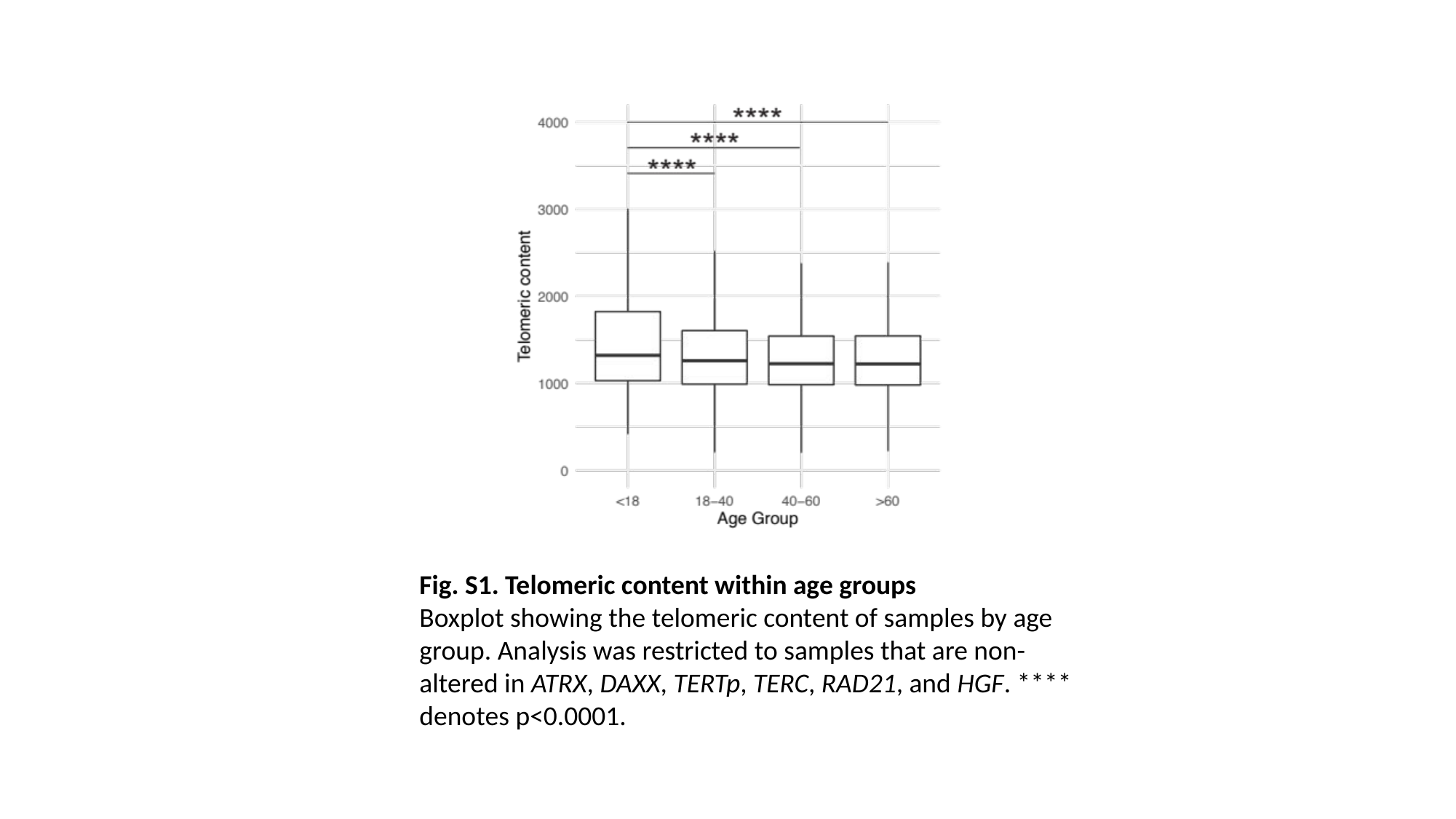

Fig. S1. Telomeric content within age groups
Boxplot showing the telomeric content of samples by age group. Analysis was restricted to samples that are non-altered in ATRX, DAXX, TERTp, TERC, RAD21, and HGF. **** denotes p<0.0001.

## Slide 2
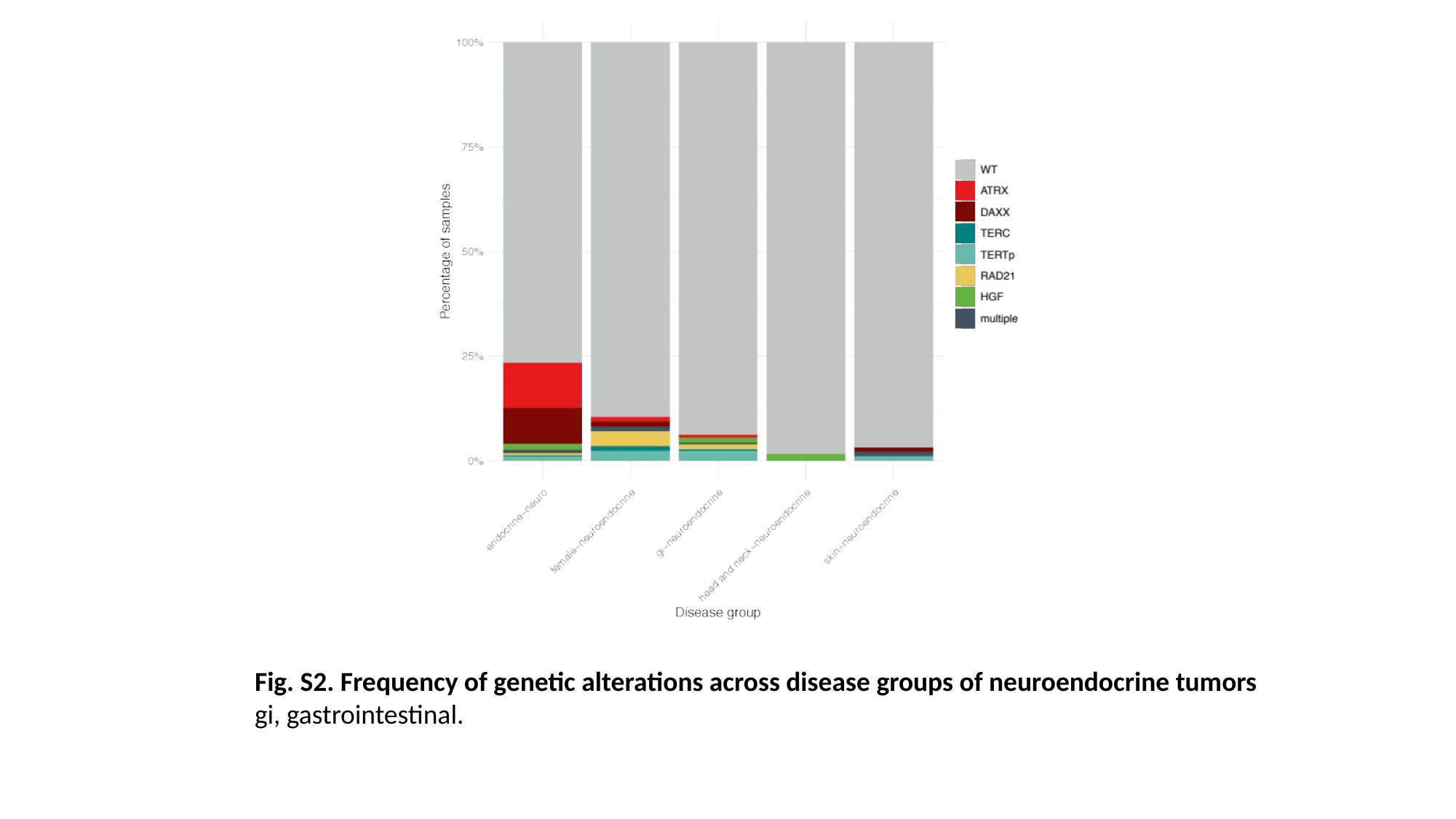

Fig. S2. Frequency of genetic alterations across disease groups of neuroendocrine tumors
gi, gastrointestinal.

## Slide 3
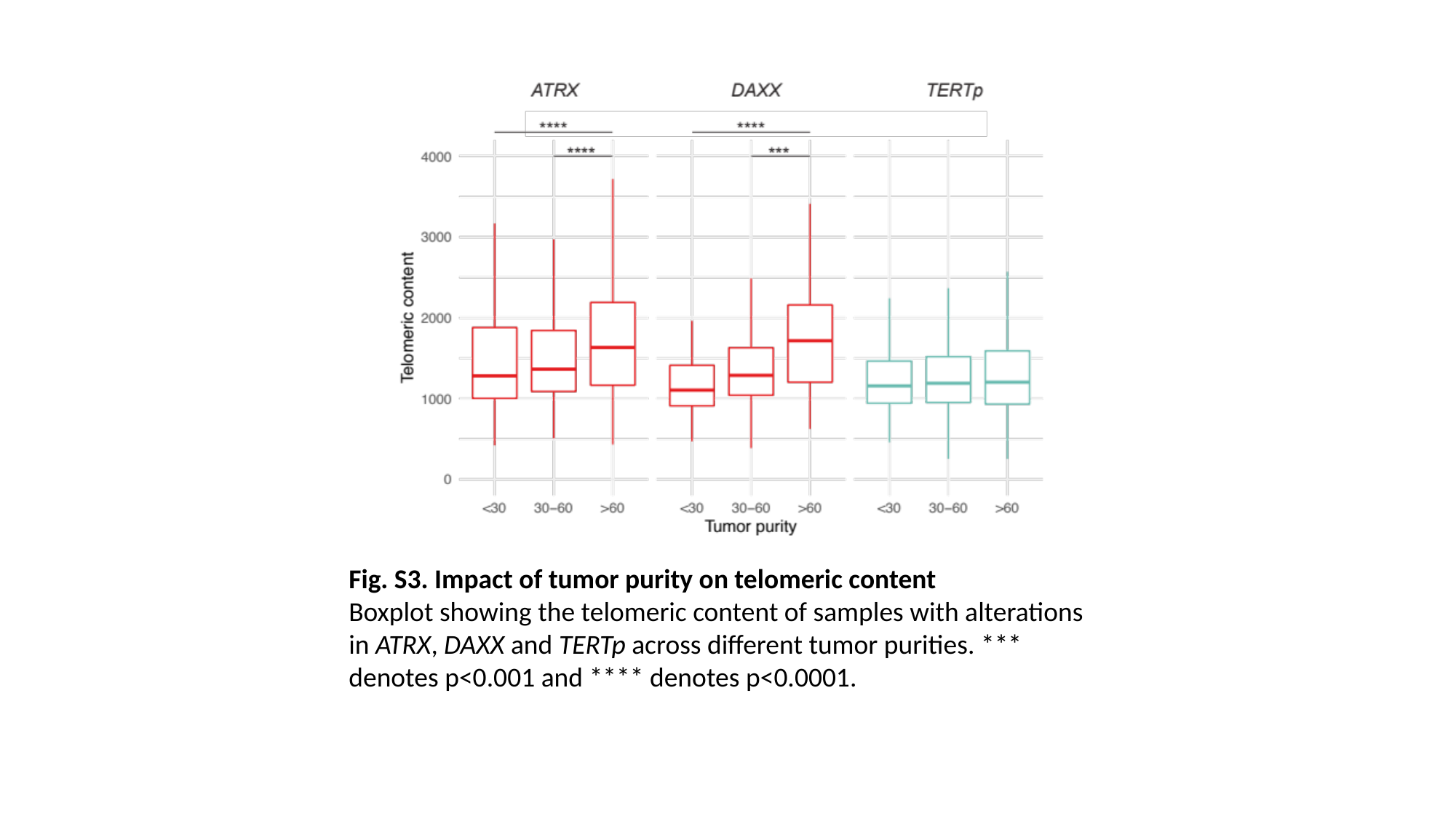

Fig. S3. Impact of tumor purity on telomeric content
Boxplot showing the telomeric content of samples with alterations in ATRX, DAXX and TERTp across different tumor purities. *** denotes p<0.001 and **** denotes p<0.0001.

## Slide 4
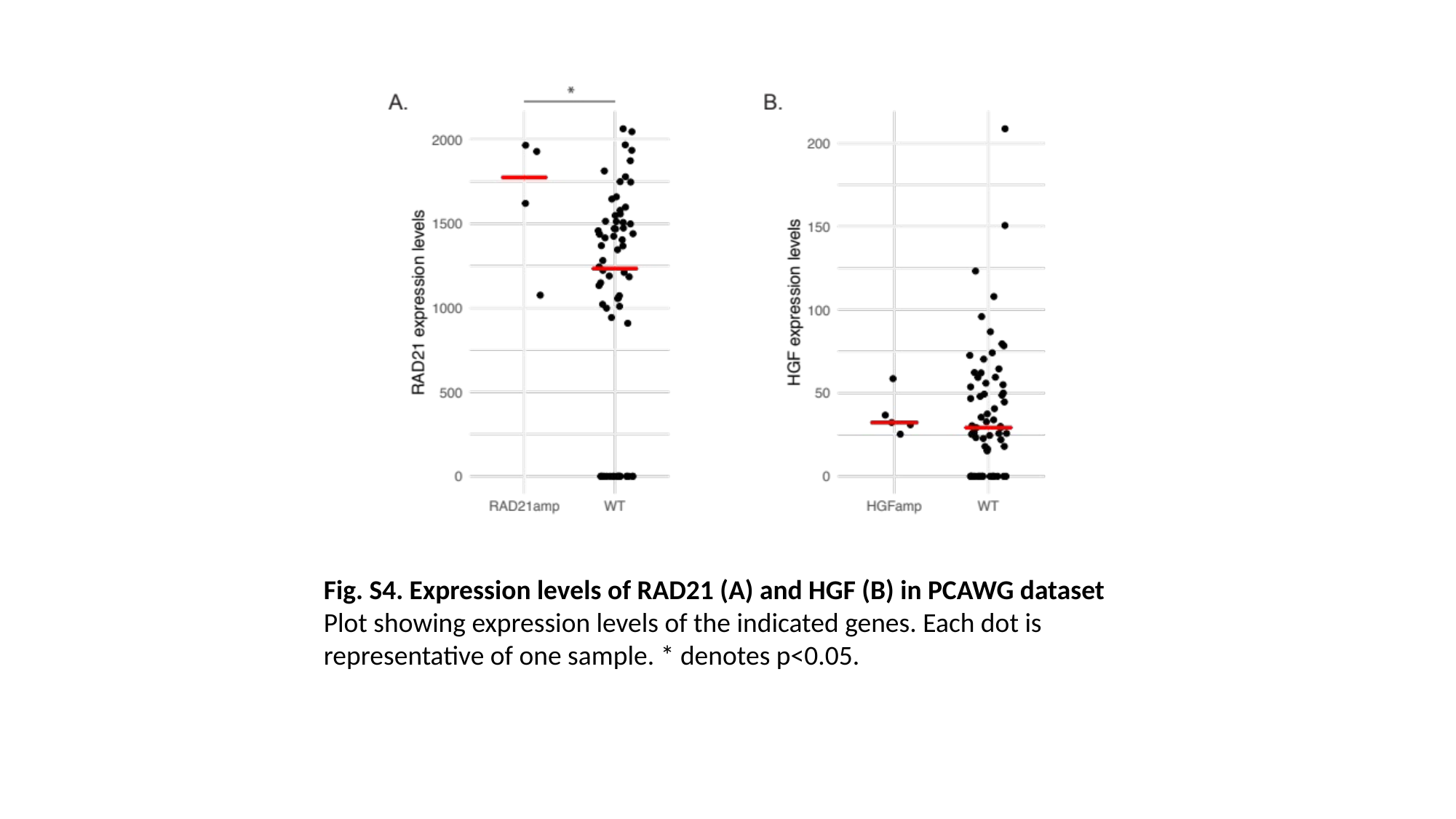

Fig. S4. Expression levels of RAD21 (A) and HGF (B) in PCAWG dataset
Plot showing expression levels of the indicated genes. Each dot is representative of one sample. * denotes p<0.05.

## Slide 5
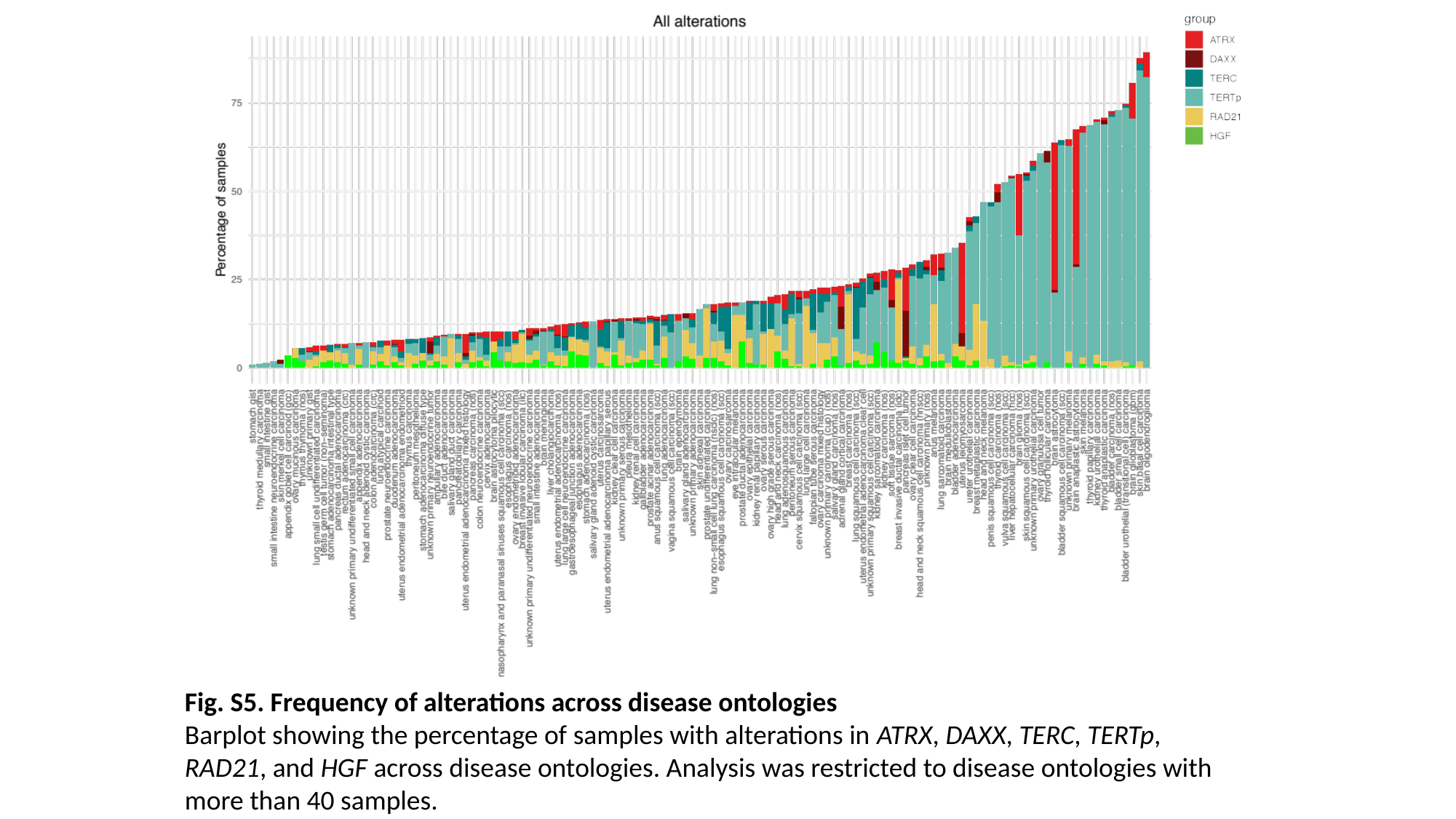

Fig. S5. Frequency of alterations across disease ontologies
Barplot showing the percentage of samples with alterations in ATRX, DAXX, TERC, TERTp, RAD21, and HGF across disease ontologies. Analysis was restricted to disease ontologies with more than 40 samples.
